# Acute Hemorrhagic Leukoencephalitis (AHLE): A Comprehensive Review on Causes, Symptoms, Link with COVID-19, Diagnosis, and Treatment

**DOI:** 10.1155/2022/6008375

**Published:** 2022-11-15

**Authors:** Mona Alromaihi

**Affiliations:** Department of Pediatrics, College of Medicine, Qassim University, Buraydah 51452, Saudi Arabia

## Abstract

Acute hemorrhagic leukoencephalitis (AHLE), also called Hurst disease, is a rare demyelinating disease of the central nervous system (CNS) marked by rapid progression and acute inflammation of the white matter. Due to the correlation in their suspected postinfectious autoimmune pathogenesis, it is regarded as the most severe form of acute disseminated encephalomyelitis (ADEM). Because this clinical scenario has a high mortality rate, aggressive and immediate treatment is required. Although the exact cause of AHLE is unknown, it usually occurs after a bacterial or viral infection, or, less frequently, after a measles or rabies vaccination. AHLE has been reported in patients with coronavirus disease 2019 (COVID-19) as a rare but serious neurological complication. However, due to the lack of evidence-based diagnostic criteria, diagnosis is difficult. The small number of cases described in the literature, which most likely reflects underreporting and/or low incidence, necessitates greater public awareness. Increased clinical suspicion and early imaging identification of this entity may allow clinicians to pursue more aggressive treatment options, potentially reducing fatal outcomes. This study focuses on symptoms and causes of AHLE, difference between AHLE and ADME, diagnosis and treatment of AHLE, and its link with COVID-19.

## 1. Introduction

Acute hemorrhagic leukoencephalitis (AHLE) is a rare demyelination disorder that causes rapid neurologic deterioration and death. Although its precise pathophysiology is unknown, it is most commonly described as a postinfectious complication of an upper respiratory illness [1, 2]. AHLE is a severe form of ADEM. AHLE has a higher rate of morbidity and mortality than ADEM, but it is much less common. AHLE is likely to be underreported and underrecognized due to the disease's rarity and a lengthy diagnostic procedure [3, 4].

AHLE is also known as Weston-Hurst syndrome or Hurst disease. Weston-Hurst was the one to describe this syndrome in 1941, when he reported two adults who developed acute encephalopathy with focal neurologic signs following a respiratory infection. The first patient (house wife) died two days after developing aphasia, headache, and right-sided hemiparesis, and the second patient (munitions worker) died one week after developing aphasia, confusion, seizures, and going into a coma. The pathologic lesions seen in the two patients were similar, with edema; hemorrhagic white matter lesions, primarily limited to one cerebral hemisphere; demyelination; perivascular polymorphonuclear infiltrates; necrosis; and fibrin deposition in and around blood vessels [5, 6].

AHLE is a type of brain inflammatory disease that primarily affects the cerebrum, but can also affect the brain stem, cerebellum, and spinal cord. The condition is thought to be a hyperacute autoimmune reaction triggered by a cross-reaction between the patient's own CNS tissues and antigens from an infectious agent. It is a rare CNS acute inflammatory myelinopathy that causes progressive loss of consciousness leading to focal neurological dysfunction and coma. AHLE is distinguished by an acute onset and rapid progression of inflammation, with symmetrical, multifocal brain lesions associated with acute edematous necrosis and hemorrhage [7]. This rare disease primarily affects young adults and is frequently linked to previous (1–4 weeks) respiratory tract infections. The overall mortality rate of AHLE is high, and survivors often have neurological deficits. Death is common in the first week of the disease, with an estimated 70% mortality rate [8].

## 2. Epidemiology

The majority of the initial reports came from the Australia and United Kingdom. The following descriptions came from the Belgium, United States, France, Eastern Europe, Germany, Japan, and Turkey. Men are affected twice as likely as women [9].

## 3. Pathology, Experimental Models, and Pathogenesis

The presence of inflammation with tissue damage or perivenous demyelination is a characteristic feature of AHLE, similar to ADEM. The degree of lymphocytic infiltration, on the other hand, is less pronounced overall. There are numerous granulocytes present, as well as small perivascular hemorrhages and fibrinoid necrosis on the walls of some veins, in contrast to ADEM [10].

AHLE was initially thought to be a subtype of ADEM with a more severe disease course and aggressive lesions. This viewpoint is supported by the observation in experimental animals that autoimmune encephalomyelitis can progress to a very severe aggressive variant known as hyperacute autoimmune encephalitis [11]. Its pathology is similar to that of AHLE. Methods for inducing hyperacute experimental autoimmune encephalomyelitis (EAE) in animals include the changes in the immunization protocol (e.g., immune-stimulating adjuvant selection), use of specific animal strains (genetic background), or immune system manipulation prior to immunization. However, the pathogenic backgrounds of ADEM and AHLE in humans may differ. The most common association of AHLE with influenza virus infections is distinct from the range of infectious diseases associated with ADEM [12]. Recent genetic studies have revealed that genetic changes in the target tissue or immune system may influence the infection-triggered inflammatory response in AHLE. Genetic polymorphisms in a gene coding for the complement factor 1 were discovered in a family with two children affected by AHLE [12]. Complement is involved in immune-mediated tissue injury and inflammation, and its stimulation has been linked to hyperacute EAE lesions, so this link could explain why these patients have such severe inflammatory CNS disease. A number of other studies have found a link between common viral infections (primarily parainfluenza and influenza), ANE, and mutations in the RAN-binding protein 2 gene (RANBP2) [13]. RANBP2 is a nuclear pore protein whose dysfunction causes energy deficit in tissues infected by acute infections. The exact mechanism by which these mutations affect inflammatory mechanisms is unknown, these findings suggest that AHLE may be caused by infection-triggered (potentially autoimmune) inflammatory response that is enhanced by the dysfunction of genes responsible for the regulation of immune-mediated tissue damage [14].

## 4. Causes and Clinical Manifestations

The onset of AHLE is sudden and, in some cases, violent. It manifests between 2 days and 2 weeks after a 3 to 5-day premonitory illness. It is most common in the winter and autumn. The most common antecedent (32–35%) is viral upper respiratory infections, but there is mostly no prodrome (18%) [5]. In one-third of cases, the upper respiratory infection is followed by a period of symptom-free time before neurologic symptoms appear. Patients in hematologic remission from acute myeloid leukemia may be especially vulnerable to this rare condition (2%) [15]. Two-thirds are men. The most common ages are 20 to 40 years, but children and the adults have also been affected. Twelve percent have preexisting auto immune diseases [5].

The course is usually short and ends with disability or death. Headache, fever up to 106°F, photophobia, meningeal irritation, neck stiffness, confusion, and lethargy last several days and are followed by a coma that deepens over 4 to 10 days. It is possible to do a biphasic course over several weeks. High-dose steroids were withdrawn before the second episode in one case of biphasic disease, possibly leading to recrudescence [16]. The diffuse and massive necrosis of CNS white matter causes a wide range of neurologic symptoms. Visual field disturbances, gaze preferences, pseudobulbar palsy, aphasia, or mutism may appear to be caused by a cortical disturbance, but they are actually caused by white matter lesions undercutting cortical connections. Early signs of incontinence and motor and sensory disturbances appear [17]. Toes are usually upgoing, and reflexes are usually hyperactive. 50% of the patients have hemiparesis. Seizures or status epilepticus, involuntary movements, and dysarthria affect a smaller proportion of the population. One-third of people have papilledema, a symptom of high intracranial pressure. Death occurs as a result of herniation and brain swelling. Cerebrospinal fluid (CSF) pleocytosis resolves after a few weeks, and clinical symptoms improve over a few weeks to months [18].

Clinically, most patients with AHLE suffer from acute encephalopathy, which rapidly worsens within a few days. Almost half of those affected die as the disease progresses, and those who survive have serious neurological consequences. In most cases, laboratory tests show elevated levels of inflammatory markers such as C-reactive protein (CRP), D-dimer, procalcitonin, and serum ferritin [5, 19].

**Figure 1:**
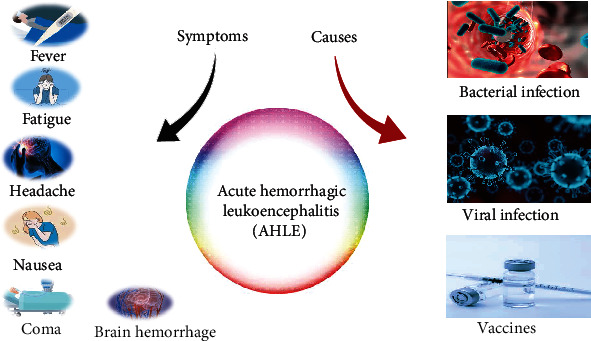
Symptoms and causes of AHLE.

Fever, headache, fatigue, nausea, neck stiffness, vomiting, seizures, or coma are common symptoms in patients diagnosed with AHLE (Figure 1). There is also evidence of brain hemorrhage resulting in white matter damage. Symptoms usually appear 2 to 12 days after a nonspecific upper respiratory infection or vaccination [20]. In addition to these symptoms, patients with AHLE may have other comorbid medical conditions such as:

(i) Respiratory infection

(ii) Abdominal pain

(iii) Focal neurologic signs

(iv) Encephalopathy

(v) Photophobia

(vi) Gaze deviation

(vii) Motor weakness

(viii) Altered state of consciousness

(ix) Sensory changes

(x) Aphasia in older patients

The disease is aggressive and severe, with patients progressing from early localized neurological symptoms to seizures and coma within days [21, 22]. Fever is usually associated with acute neurological disease. Untreated patients have a very high mortality rate. A clinical evaluation one month after onset helps determine the prognosis for long-term outcomes in surviving patients. [21]. MRI changes are similar as seen in ADEM, but with more severe brain edema and, unlike ADEM, petechial hemorrhages are present. Increased protein levels and leukocyte counts in the cerebrospinal fluid (CSF) indicate inflammation, but no oligoclonal bands or intrathecal immunoglobulin synthesis are found [10].

Although the underlying cause of AHLE is uncertain, researchers have discovered a correlation between the onset of symptoms and bacterial or viral inflammation (Figure 1). Hence, it is thought that AHLE is the result of an autoimmune response to a bacteria or virus [23]. Numerous viruses and bacteria have been linked to ADEM/AHLE (Table 1).

## 5. Pathophysiology

Demyelination occurs when human myelin antigens react with bacterial or viral antigens, resulting in the collapse of the nerve fibers' protective unit. Because autoimmune processes usually target the CNS rather than the peripheral nervous system, this immune response is unusual. Both areas of the nervous system are targeted in the case of AHLE [25]. The autoimmune process is thought to be initiated by molecular mimicry between antigens of the bacterial or viral pathogen and myelin antigens. Myelin basic protein peptides have been found to have homologies with viruses such as influenza, measles, adenovirus, hepatitis B virus, and Epstein-Barr virus. Sensitivity to this type of autoimmunity is predominant, as some molecules of the major class I and II histocompatibility complex are more effective in presenting homologous peptides for T cell activation than others may be affected by haplotypes of compatible gene complexes. The disease's rarity is probably due to predisposing genetic factors. Studies linking post vaccination encephalomyelitis caused by the Semple rabies vaccine to human leukocyte antigen-DR17 and -DR9, as well as human leukocyte antigen-DRB1^∗^01 and -DRB1^∗^03(017) with ADEM in the Russian population, implying a genetic link [26].

In a recent study, researchers found support for this hypothesis by injecting a Theiler's murine encephalomyelitis virus into mice. In the experiment, CD8+T cells were activated in C57BL/6 mice, resulting in hemorrhagic demyelination in 24 hours. This was the first time an AHLE murine model had been shown [27].

Meanwhile, several studies have found a tenuous link between the disorder and measles, rabies, or vaccination; however, further research is required to confirm this link [20, 23].

## 6. AHLE and ADEM

AHLE and ADEM share many features. This is because both are usually preceded by upper respiratory tract infections, or EBV, HSV, mumps, mumps, influenza, rubella virus infections, or vaccination. Despite the fact that many infectious diseases and vaccine precursors are associated with both diseases, active research into these causes is often futile. AHLE is more commonly diagnosed in young adults than ADEM, which is more commonly diagnosed in children. AHLE may be more common in people of Pacific Island ancestry and/or Asian, but ADEM does not affect people of any ethnicity specifically [25, 28].

It is unclear whether ADEM and AHLE are part of a series of diseases with the same underlying process resulting from an autoimmune response caused by prodromal infection, or if they are separate entities. However, different types of CNS infiltrates, such as neutrophils in AHLE and lymphocytes in ADEM, do not support the idea of spectrum diseases. Many autoimmune response mechanisms and histopathologic features are similar in AHLE and ADEM. Hence, AHLE is regarded as the most severe subtype of ADEM [28].

Because ADEM and AHLE are on the same disease spectrum, there is no clear distinction between the two. Hence, distinguishing the two with MRI is difficult. White matter lesions in both diseases can be caused by edema and inflammation at first, and then by axonal loss and demyelination later on [29]. ADEM has less cerebral edema, smaller lesions, and rare hemorrhage. Further distinction is that in AHLE, the subcortical U fibers are usually spared. AHLE produces a predominantly neutrophilic infiltrate with pericapillary ball and ring hemorrhages and hematomas, whereas ADEM produces a predominantly lymphocyte-rich infiltrate [30].  Table 2 compares AHLE and ADEM in terms of neuroradiologic, clinical, and laboratory differences.

## 7. Diagnosis

AHLE is diagnosed primarily through neuroimaging, CSF analysis, and histopathology. Due to the lack of formal research, guidelines for defining diagnostic algorithms are absent and cannot be derived from current data. Furthermore, most clinical scenarios described in the literature include signs and symptoms that lead clinicians to suspect ADEM. Individual clinical features do not make a definitive distinction between ADEM and AHLE, but the coexistence of multiple signs and symptoms helps to distinguish between these two entities. Given that the clinical course and syndromic appearance of ADEM and AHLE are frequently indistinguishable, treatment options are similar, and the body of evidence for the latter is limited for both entities, the need for a reliable differentiation appears dubious [5].

Clinical examination and, more importantly, CT scans, CSF studies, brain biopsy, and MRIs can be used to make a diagnosis [23, 31]. CSF tests for increased protein levels and leukocytosis are usually positive, but polymerase chain reaction testing and infectious cause cultures are usually negative. A head CT scan may reveal hypodensities in the affected grey and white matter. Hyperintense T2-weighted lesions will be visible on a brain MRI. Better visualization will be provided by the FLAIR sequence [31]. A brain biopsy may reveal pathognomonic findings such as demyelination, hemorrhagic necrosis, and perivascular infiltrates. Although direct brain tissue examination is the most accurate method of diagnosing AHLE, this might not be possible in all patients [23]. Clinical diagnosis can be made based on the severity of the symptoms, the disease's clinical course, and radiological findings. The radiological findings of ADEM and AHLE are similar [23] [24].

AHLE is histopathologically characterized by necrosis and perivascular microhemorrhages, demyelination with fibrin exudate, and axonal injury. Perivascular inflammation is of variable severity, but is usually neutrophilic with lymphocyte and macrophage infiltration [32, 33]. Many people die as a result of edema and massive hemorrhage caused by herniation. Finally, immunosuppression and cerebral edema management are the treatment goals [34].

MRI is useful in the early detection of AHLE, which is characterized by white matter tracts with hemorrhage on SWI and sparing of grey matter. The changes are usually multiple, with the parietal and frontal lobes most affected, but the brainstem, spinal cord, and basal ganglia can also be affected [30, 35, 36]. Brain MRI is critical because it detects confluent white matter lesions with space-occupying effects, significant edema, and petechial hemorrhages. The lesions' location appears to be highly variable. Nearly two-thirds of patients have uni- or bilateral hemispheric involvement, but other distributional patterns have been described. The presence of intraparenchymal hemorrhages on cerebral MRI, however, is the most useful feature in distinguishing AHLE from ADEM [26, 37].

## 8. Management and Treatment

The onset of AHLE symptoms occurs concurrently as the disease progresses, making it an extremely difficult condition to treat. Despite this, many current studies and clinical trials are focused on developing effective and long-term treatments for the disease [23].

No controlled studies or large case series have been published to help guide the management of this often fatal and rare condition. Management is based on expert opinion, knowledge of the underlying pathology, and isolated case reports data. Several case reports have been published reporting positive results from a variety of immunosuppressive methods, including intravenous immunoglobulins, high-dose corticosteroids, cyclophosphamide, and plasmapheresis [30]. Seales and Greer described how they used mannitol, hyperventilation, and phenobarbital to treat a patient with high intracranial pressure. The patient in that case had no long-term neurological effects. It has also been discussed how to treat raised intracranial pressure surgically [18, 33].

Treatment for AHLE has the potential to alter the disease's prognosis. The first line of treatment is aggressive ICP control, which may necessitate decompressive craniectomy in some cases. The majority of patients, however, can be treated medically [8]. Immunomodulation therapy is used in the second stage of treatment to deal with the persistently high levels of inflammation. Although no treatment guidelines for AHLE have been established, multiple studies and case reports suggest that IVIG, high-dose steroids, and plasmapheresis should be used as the primary treatment [23, 31, 33]. On initial presentation, a pulse dose of IV methylprednisolone, along with acyclovir and antibiotics, should be started. It should be taken for 3-5 days, then followed by 4-6 weeks of oral prednisolone [38]. Several case reports and small studies have demonstrated the efficacy of daily IVIG therapy, either as a single dose or as a five-day course. Despite the fact that no clinical trials comparing plasmapheresis and IVIG for the treatment of AHLE have been conducted, evidence from multiple series and case reports suggests that plasmapheresis should be used when steroid and IVIG fail [39].

Previous studies have reported monophasic disease with a good prognosis when the first attack is overcome [30]. There have been no reports of AHLE recurrence. It is also unclear whether the presence or absence of previous respiratory disease has prognostic value. AHLE is not only a diagnostic but also a therapeutic challenge, as treatment guidelines for AHLE are not well-established. Further research is needed to better characterize this rare disease and provide more information about treatment and prognosis options.

## 9. AHLE and COVID-19

COVID-19, which is caused by the severe acute respiratory syndrome coronavirus 2 (SARS-CoV-2), has been linked to neurological problems in a number of studies. [40, 41]. Most associated neurologic dysfunctions are mild symptoms such as dyspepsia and anosmia, but severe debilitating neuropathies such as meningoencephalitis and stroke are rare [42]. These symptoms are most likely caused by SARS-CoV-2 infecting the brain directly through olfactory neuroepithelium infection, or by circulating monocytes and lymphocytes that can cross the blood-brain barrier [43, 44]. Endothelial cells, neurons, glia, smooth muscle, and vascular pericytes all express the angiotensin-converting enzyme 2 (ACE2) receptor in the brain and spinal cord, which is the primary receptor for SARS-CoV-2 spike protein [45–47]. RT-PCR, immunohistochemistry (IHC), electron microscopy, and in situ hybridization (ISH) were all used for the detection of SARS-CoV-2 in the olfactory epithelium, brain, and cranial nerves [48–50]. Neuropathological findings such as microglial nodules, parenchymal lymphocytic and leptomeningeal inflammation, and neuronophagy are also consistent with a viral meningoencephalitis diagnosis. On hematoxylin and eosin- (H&E-) stained slides, however, neither viral inclusions nor cytopathic changes could be seen. Furthermore, in the several studies that have reported evidence of protein or viral RNA in the brainstem or cranial nerves, the distribution and degree of neuropathologic changes have shown no correlation with the amount of virus in a given area of pathology, implying that pathology is inferior to the systemic effects of viral infection. Evidently, convincing evidence has been found that systemic hypercoagulability is important in the COVID-19-related stroke process, as SARS-CoV-2 could elicit endotheliopathy and cytokine storm, resulting in histologic findings of microthrombi, hemorrhages, and infarcts [2, 51–53].

However, whether parainfectious, autoimmune-mediated processes such as ADEM or its hyperacute form AHLE can sincerely be attributed to COVID-19 remains debatable. One source of contention is the scarcity of neuropathological descriptions of ADEM and AHLE, despite the fact that a few clinical and imaging case studies of COVID-19 patients have revealed lesions characterization of ADEM or AHLE [54–57]. Only a few case reports with ADEM and AHLE neuropathological features in adult deaths from COVID-19 complications have been published so far. Another source of debate is whether the ADEM-like or AHLE-like pathology observed is the result of a basic demyelinating process, a secondary white matter lesion caused by coexisting vascular disease, or a combination of the two [58–60].

Despite the fact that patients with COVID-19 often have neurological symptoms, only a few cases of AHLE related to COVID-19 have been recorded so far [61–66]. The neurological symptoms of COVID-19 are thought to be secondary to direct viral cytotoxic effects on neurons, immune inflammation, and the development of intracranial cytokine storms [64, 67]. Recent studies show that AHLE patients have significantly higher cytokine levels than ADEM patients or noninflammatory neurological conditions [67]. SARS-CoV-2 can cause direct endothelial damage and/or secondary inflammation, as evidenced by vasculitis-like symptoms in some patients [68]. Demyelination, fibrinoid necrosis, perivascular inflammatory infiltrates, necrotizing vasculitis, and hemorrhages are all pathological features of AHLE [50]. Confined neuropathological data in COVID-19 patients show similar features in the absence of demonstrable infectious pathogens, suggesting a quasiinfectious causative mechanism [69, 70].

AHLE is characterized by undefined multifocal lesions of variable size (generally larger than 1 cm) of white matter, including the cerebral hemisphere, mainly the occipital and parietal lobes. There is a predominance of subcortical and deep white matter, with a distinctive asymmetric distribution. Although less common, brainstem, deep grey matter, and cerebellar peduncle involvement can occur [62, 71]. FLAIR and T2W images show hyperintense lesions, T1W images show hypointense lesions, and susceptibility-weighted images (SWI) show microhemorrhage-related blooming. Diffusion and contrast characteristics vary. Widespread microhemorrhages and extensive brainstem involvement are often indicators of a poor prognosis [72–74]. The presence of unifocal pseudotumoral lesions including the brainstem and corpus callosum is unusual [64, 75, 76].

Leukoencephalopathy and microhemorrhages-related changes, on the other hand, are common in critically ill COVID-19 patients with or without AHLE or ADEM clinical features. Hypoxia, sepsis, direct infection, metabolic causes, and PRES are all suspected causal factors in these patients [77]. However, imaging features favoring the presence of white matter changes with gross hemorrhage or florid micro causing a mass effect is an indication of AHLE [5].

To conclude, a COVID-19 patient with rapid neurological deterioration should prompt the treating physician to suspect AHLE, perform neuroimaging to confirm a compatible lesion pattern, and treat aggressively. Several discrepancies between ADEM and AHLE after COVID-19 infection and ADEM and AHLE without a history of prior COVID-19 infection were identified by Manzano et al. in 2021. According to Manzano et al., the great majority of recorded cases involve adults rather than children. The previous systemic infection was usually severe rather than trivial. On neuroimaging, hemorrhagic changes within demyelinating lesions were prevalent. Despite appropriate immunotherapy, the outcome was poor [78].

High-dose IV steroids, typically methylprednisolone, remain the first-line treatment [5]. Plasma exchange and intravenous immunoglobulin (IVIG) can be used to treat refractory cases. According to a recent research of cytokines in COVID-19, tocilizumab and broad-spectrum immunomodulators are recommended for the first suspicion of AHLE [19, 67]. The treatment of COVID-19-related AHLE is still a new area and cannot be improved without more experience and knowledge of the disease.

## 10. Prognosis and Complications

The prognosis for AHLE is poor, with death usually occurring within a week of onset due to increased intracranial pressure (ICP) control. Psychiatric symptoms, mental deterioration, and seizures are common in survivors. Only a few patients have fully recovered [18, 33].

## 11. Conclusion

AHLE is a rare and rapidly fatal disease with an unknown etiology. There is a scarcity of literature on the management and presentation of this rare disease. Diagnosis is difficult because the level of evidence regarding the diagnostic yield of neuroimaging, clinical, and laboratory characteristics is still very low. Future research should concentrate on treatment characteristics and their impact on the course and outcome.

## Figures and Tables

**Table 1:** Bacterial and viral pathogens commonly associated with AHLE [24].

Bacteria	*Mycoplasma pneumonia*
	*Borrelia burgdorferi*
	*Leptospira*
	*Streptococcus*

Viruses	Influenza virus
	Enterovirus
	Measles
	Mumps
	Rebella
	Varicella zoster
	Epstein Barr virus
	Cytomegalovirus
	Herpes simplex virus
	Hepatitis A
	Respiratory syncytial virus
	John Cunningham virus
	Eastern and western equine viruses
	West Nile
	California
	SARS-CoV-2

**Table 2:** Neuroradiologic, clinical, and laboratory differences between AHLE and ADEM [5].

Characteristics	AHLE	ADEM
Age group affected	Young adults	Children
Epidemiology	Incidence unknown Male predominance	Incidence 0.3–0.6 per 100,000 per year (i) Male predominance (ii) More common in children and teenagers

Triggering event	Respiratory tract infections	Respiratory tract infections, viral exanthema, and vaccination

Clinical findings	Focal neurological symptoms according to the location of lesions (i) Symptoms and signs of elevated intracranial pressure possible	Focal neurological symptoms according to the location of lesions (i) Unspecific encephalopathy
Symptoms		

Clinical course	Fulminant, frequently evolving to coma/death within days	Less fulminant, coma unusual

Onset	Hyperacute, fulminant	Acute

Blood picture	Leukocytosis	Normal

Radiological findings	Larger lesions, confluent (i) Significant edema with space-occupying effect (Petechial) hemorrhages	Hyperintense lesions of the white matter No data
FLAIR		
SWI/T2^∗^		

Histological and laboratory findings CSF	Granulocytic pleocytosis (i) Elevated protein in 87% (ii) Normal glucose (iii) Possibly erythrocytes/ferritin	Protein increased in 23–62% of pediatric Patients Lymphocytic pleocytosis

Peripheral blood histopathology	Neutrophil-predominant leukocytosis Necrosis of small vessels (i) Perivascular fibrin exudation (ii) Hemorrhages (“ring-and-ball”) (iii) Infiltration with neutrophils and macrophages (iv) Demyelination in later stages	No leukocytosis Perivascular demyelination with lymphocytic Infiltration

Pathology	Demyelination, ring hemorrhages, and fibrinoid necrosis	Demyelination, Perivenular inflammation

ESR/acute phase reactants	Elevated	Normal

CSF cellularity	Red blood cells, neutrophils	Mononuclear

Urine	Occasionally proteinuria	Normal

Neuroimaging	Large lesions, hemorrhage, necrosis, and mass effect	Multifocal white matter lesions

Treatment options	(i) Glucocorticoids (ii) Plasmapheresis (iii) IVIG (iv) Rituximab, cyclophosphamide	(i) IVIG (ii) Glucocorticoids (iii) Plasmapheresis

Outcome	Poor	Generally good

## Data Availability

The data presented in this study are available within the article.
